# Understanding the complex interplay between tau, amyloid and the network in the spatiotemporal progression of Alzheimer’s Disease

**DOI:** 10.1101/2024.03.05.583407

**Published:** 2024-03-11

**Authors:** Ashish Raj, Justin Torok

**Affiliations:** 1 Department of Radiology, University of California at San Francisco, USA; 1 Irving St, AC-116, Box 028, Parnassus Campus, San Francisco, CA 94122; 2 Bakar Computational Health Sciences Institute, UCSF

## Abstract

It is well known that Aβ and tau proteins are deposited stereotypically in brain regions to cause Alzheimer’s disease. The interaction of amyloid and tau in neurodegenerative diseases is a central feature and key to understanding AD pathophysiology. However their mechanisms are controversial, and many aspects do not fit current theories that rely on cell-autonomous factors. While cell culture and animal studies point to various interaction mechanisms between amyloid and tau, their causal direction and mode (local, remote or network-mediated) remain unknown in human subjects. Further, cross-protein interaction is yet to be reconciled with canonical observations that the two species do not co-localize significantly either in space or in time, and do not target the same neuronal populations. To answer these questions quantitatively, in this study we employed a mathematical reaction-diffusion model encoding the biophysical mechanisms underlying self-assembly, trans-neuronal network propagation and enzymtic cross-species coupling of amyloid and tau. We first established that the spatiotemporal evolution of theoretical tau and Aβ correctly predicts empirical patterns of regional Aβ, tau and atrophy. Remarkably, the introduction of a 1-way Aβ→tau interaction was critical to the models’ success. In comparison, both the non-interacting and the 2-way interaction models were significantly worse. We also found that network-mediated spread is essential; alternative modes of spread involving proximity or fiber length fare much worse. This mathematical exposition of the “pas de deux” of co-evolving proteins provides crucial quantitative and whole-brain support to the concept of amyloid-facilitated-tauopathy rather than the classic amyloid-cascade or pure-tau hypotheses, and helps explain certain known but poorly understood aspects of AD.

## INTRODUCTION

Alzheimer’s disease (AD) involves widespread and progressive deposition of amyloid beta (Aβ) protein in cortical plaques and tau tangles^1,2^. Aβ usually first appears in frontal regions and subsequently spreads to allocortical, diencephalic, brainstem, striatal and basal forebrain regions^2,3^. In contrast to Aβ, tau tangles appear first in locus coeruleus, then entorhinal cortex, followed by an orderly spread into hippocampus, amygdala, temporal lobe, basal forebrain, and isocortical association areas^1^. The dominant “amyloid cascade hypothesis”^7^, posits that amyloid is the upstream factor, whose early abnormal accumulation in the brain causes a cascade of downstream events that recruit misfolded tau. However, this hypothesis has encountered several difficulties, including the spectacular failure of many large clinical trials of amyloid-targeting therapies, and the fact that the temporal and regional distribution of Aβ is quite dissociated from that of tau^4–6^. as well as of downstream atrophy and cognitive deficits. This has led to the search of other mechanisms, especially the role of tau, which is increasingly considered more central to D pathophysiology. However, it is unlikely that tau alone can explain AD pathogenesis, due to overwhelming mechanistic, genetic and demographic evidence of amyloid involvement.

Among potential alternative concepts put forward to reconcile these difficulties, a prominent one is the emerging concept of network-based transmission of both amyloid and tau. Evidence is accumulating in favor of self-assembly and trans-neuronal propagation of amyloid and tau, indeed, of all neurodegenerative pathologies^19–22^. Unlike conventional assumptions about spatial spread of pathology in the brain, this emerging concept implies spread along axonal projections, and the resulting networked spread has repeatedly been substantiated by neuroimaging^9,10,23,24^. Network-mediated spread of amyloid and tau offers an attractive means of reconciling above difficulties: the two species may interact locally, but the effects of these interactions do not remain local, and may propagate across neural circuits to distant regions. Given that the two species have unique and different “epicenters” or seeding loci, this would lead to the apparent observation of their deposition in separate non-overlapping regions. Thus, the cross-species interaction, in the background of network-mediated propagation, may present a plausible framework for understanding AD progression.

Many experimental and mechanistic studies are available that point to a complex interaction between amyloid and tau. It is well known that Aβ facilitates the aggregation of tau and influences the course and severity of downstream atrophy^16–18^. Mechanistic studies point to various modes of interaction^17,30–32^.

While these studies provide important mechanistic evidence in model organisms, they may be sensitive to idiosyncratic methodological choices, leading to a diversity of mechanistic possibilities that may not generalize and may not be germane to human disease. Key aspects of brain-wide propagation of tau and amyloid, and the exact mechanistic form their mutual interactions, therefore remain unsupported empirically in human AD. Since experimental testing of mechanistic hypotheses is difficult in humans, here we took a somewhat unorthodox approach, by relying on a *computational* rather than *experimental* interrogation of the mechanistic interaction between tau and amyloid. We first formulated a mathematical model that recapitulates the spatiotemporal evolution of human AD, based on recent advances in the mathematical modeling of reaction-diffusion or network processes that have emerged as a powerful means of evaluating the brain-wide consequences of biophysical mechanisms underlying self-assembly and propagation of neurodegenerative pathologies^8–15^. On top of this base network model, we then built various *interaction models* that allow Aβ and tau species to enzymatically interact in local neural populations. We compared the performance of each interaction model via thorough statistical adjudication, by accumulating model evidence on large neuroimaging (tau and amyloid PET, MRI) datasets of AD spectrum subjects. We devised a fitting procedure to obtain model parameters that best match individual subjects’ regional disease patterns. In this manner we were not only able to determine the most well-supported modes of cross-species interactions, but also their applicable kinetic rates. Although spatial and network spread models of single pathological species have been previously reported^18–15^, and data-driven models of multiple biomarkers are also available^15,28,29^, this study is unique in modeling and evaluating directly on empirical data the network-mediated transmission and interaction of tau and amyloid *jointly*.

We show that both network propagation and amyloid-tau interaction are necessary to recapitulate human AD data. Our data conclusively support that network-mediated spread, with 1-way interaction, whereby amyloid facilitates local tau aggregation, is the most parsimonious and accurate model, yielding correlations above 0.7 against empirical tau topography. Other interactions, including bidirectional ones and those involving enhanced pathology spread instead of aggregation, were not well supported on statistical tests. In totality, this “toxic *pas de deux*” of co-evolving tau and amyloid pathologies provides critical numerical support to mechanistic hypotheses not possible to be tested directly in humans. Our computational testbed may become an important future tool for the generation and testing of novel mechanistic hypotheses with the potential to obviate expensive and time-consuming mechanistic studies in model organisms.

## MATERIALS AND METHODS

### Mathematical Model of Network Transmission

#### Notation.

Brain’s anatomic connectivity network is defined on the graph 𝒢 = {𝒱, ℰ} whose nodes *v*_*i*_ ∈ 𝒱, *i* ∈ [1, *N*_*roi*_] represent grey matter structures, and edges *e*_*i*,*j*_ ∈ ℰ represent fiber connectivity. Structures *v*_*i*_ comes from parcellation of brain MRI with *N*_*roi*_ gray matter regions, and connection strength of the edge between any pair of regions i,j, *c*_*i*,*j*_ is measured by fiber tractography^61^, giving the network connectivity matrix *C* = {*c*_*i*,*j*_, (*i*, *j*) ∈ ℰ}. Each node has an in-degree and an out-degree given by row and column sums of the matrix C: **d**_*row*,*i*_ = ∑_*j*_ *c*_*i*,*j*_, **d**_*col*,*j*_ = ∑_*i*_ *c*_*i*,*j*_. Define *H* as the graph Laplacian matrix *H* = *I* − *diag*(**d**_*row*_ **d**_*col*_)^−1/2^ *C*. This Laplacian definition normalizes each node’s connectivity by the geometric mean of its in/out-degree, to accommodate brain parcellations of varying size and connectivity, and emphasizes nodes with high imbalance between in-coming and out-going connections. We define two time-varying vectors containing levels of *Aβ* and tau at each node: **x**_*Aβ*_ (*t*) and **x**_*τ*_(*t*) respectively.

#### Non-linear reaction + network diffusion with local growth and cross-tau-Aβ interaction

Here we propose a complete and realistic model of pathology dynamics that incorporates the production, growth and spread of both Aβ and tau, as well as the interaction between them.

##### 1) **The No-interaction model**:


(1a) $$\text{Evolution of tau: }\;\frac{dx_{\tau }(t)}{dt}=-\beta Hx_{\tau }(t)+\alpha f_{\tau }(t)e_{ERC}$$

(1b) $$\text{ Evolution of Aβ}:\;\frac{dx_{A\beta }(t)}{dt}=-\beta \text{H}x_{A\beta }(t)+\alpha f_{A\beta }(t)\left(m\cdot x_{APP}\right)$$

The first term on the right side in Eqs (1) above represents network diffusion, following our prior proposal^23^, whereby pathology spread occurs by diffusion restricted along network connections. This involves the connectome’s Laplacian matrix H and the diffusivity rate constant *β*. Diffusive spread implies its rate depends both concentration gradients and the amount of connectivity between regions. This model captures trans-neuronal propagation as a connectivity- rather than distance-based process, enacted via active axonal transport followed by membrane- or exocytotic-processes into extracellular space. Fiber length does not enter this model since there is no evidence that axonal transport efficiency is dependent on fiber length. **Evolution of tau**: The second term of Eq (1a) introduces a focal seeding event, at the entorhinal cortex (EC), i.e. where all misfolded pathology is produced; for which there is historically accepted evidence that pathology begins in EC. No other region is capable of producing misfolded pathology, but is able to further transmit it. **Evolution of Aβ**: Aβ spread is modeled identically to tau, with the same global diffusivity constant *β*. Unlike tau, Aβ appears to have a diffuse production mediated by metabolism – this is encoded by the second term in Eq (1b). Since onset of production is gradual, followed by eventual decline and plateauing, we govern the production terms by long-duration gamma-shaped driving functions *f*_*Aβ*_ and *f*_*τ*_; see SI: Note 3 and Figure S2. The production of misfolded amyloid is assumed to be proportional to both its baseline metabolism **m** measured via FDG-PET of healthy subjects, as well as the region’s pool of available Amyloid Precursor Protein (APP), **x**_*APP*_, from which *Aβ* is cleaved.

#### Modeling the network-mediated interaction between tau and amyloid

Several possibilities of cross-interaction exist; here we specify those having the highest empirical or mechanistic support.

##### 2) **1-way interaction model: Amyloid affects tau aggregation**.

A new cross-species interaction term was added via coupling coefficient *γ* to simulate a potential role for amyloid to govern the rate of growth of misfolded tau ^16–18^:

(2a) $$\text{Amyloid-facilitated tau aggregation: }\frac{dx_{\tau }(t)}{dt}=-\beta \text{H}x_{\tau }(t)+\alpha f_{\tau }(t)e_{EC}+\gamma \left(x_{A\beta }(t)\cdot x_{\tau }(t)\cdot \left(1-x_{\tau }(t)/K\right)\right)$$

Note the last term has a logistic growth term of the form $x\left(1-\frac{x}{K}\right)$. This term is required to ensure that tau does not grow exponentially indefinitely, and recognizes that realistic tau growth must be limited by overall capacity of healthy tau from which toxic tau can be cleaved. The capacity level is controlled by the parameter K. The equation for amyloid evolution (**2b**) remains the same as (1b).

##### 3) **1-way interaction model: Amyloid affects tau diffusion into the network**.

In this model amyloid does not influence tau aggregation but increases the rate of tau diffusivity into thE network in areas where amyloid id high and less diffusivity in areas where amyloid is low. This model simply modulates the scalar diffusivity constant in Eq (1a) by allowing it to be regionally-varying:

(3a) $$\text{Amyloid-facilitated tau diffusion: }\frac{dx_{\tau }(t)}{dt}=-\beta \cdot \text{diag}\left\{x_{A\beta }(t)\right\}\cdot \text{H}x_{\tau }(t)+\alpha f_{\tau }(t)e_{EC}$$

In this model tau in every region experiences a different net diffusivity, depending on the local level of amyloid. The equation for amyloid evolution (**3b**) remains the same as (**1b**).

##### 4) **Tau to amyloid interaction model**.

The reverse effect of tau on Aβ production does not have firm pathophysiological support^16^. However, for completeness we also explored the alternative interaction via the coefficient *η*. The equation for tau evolution (**4a**) remains the same as (**1a**).


(4b) $$\text{Tau-facilitated Aβ}:\frac{dx_{A\beta }(t)}{dt}=-\beta \text{H}x_{A\beta }(t)+\alpha f_{A\beta }(t)\left(m\cdot x_{APP}\right)+\eta \left(x_{\tau }(t)\cdot x_{A\beta }(t)\cdot \left(1-x_{A\beta }(t)/K\right)\right)$$

##### 5) **Two-way interaction model**.

Finally we included in our analyses a bidirectional model whereby both species influence each other. Hence Eq 5a for tau evolution is the same as Eq (2a) and Eq (**5b**) for amyloid evolution is the same as Eq (4b).

##### 6) **Proximity-based spread**.

We simulated proximity-based spread using the same formulation as Eqns (1a and 1b), but replacing the connectome-derived Laplacian with adjacency matrix computed from inter-regional Euclidean distance: $C_{\text{dist }}=\exp\left(-\frac{D}{\sigma }\right)$, where *D* = {*d*_*i,j*_} is the matrix of distance between regions i,j, and *σ* is the standard deviation over all elements of D. Laplacian *H*_*dist*_ was defined analogously to the connectome Laplacian *H*, and replaced that term in Eq (1). We also evaluated a Gaussian relationship instead of exponential, but the results were generally worse and were not further evaluated. Denote the full model by Eq (**6a**, **6b**).

##### 7) **Fiber distance-driven spread**.

We simulated spread along fiber projections using the same formulation as Eq (1), but replacing connectivity with fiber distance, as per $C_{\text{fiberdist }}=\exp\left(-\frac{L}{\sigma }\right)$, where 𝐿 = {*l*_*i,j*_}. Here *l*_*i.j*_is the average length of streamlines between regions i,j. Laplacian *H*_*fi*𝑏*erdist*_ was defined analogously to the connectome Laplacian *H*. Denote the full model by Eq (**7a**, **7b**).

#### Implementation.

The above theoretical models were numerically solved using MATAB’s **ode45()** solver, jointly for both amyloid and tau, even in those cases when some parameter choices would lead to decoupled equations. Initial condition was set such that *x*_*τ*,*Aβ*_ (*t* = 0) = 0; however note that the seeding and production of the two species are non-zero, via the term *f*(⋅).

### Experimental Design

#### Subjects and data.

Data used in this study were obtained from the ADNI^33^ database (http://adni.loni.usc.edu); consisting of 531 ADNI-3 subjects who had at least one exam of all three: MRI, AV1451-PET and AV45-PET, available by 1/1/2021. Demographic information is in Table S1. These data were processed to obtain regional statistics of pathology and atrophy, the latter used in this analysis as a surrogate measure for tau tangles/neurodegeneration. Anatomic connectomes were computed from healthy diffusion MRI and tractography algorithms. The primary dataset was evaluated on the 86-region Desikan atlas and the Supplementary dataset on the 90-region AAL atlas, using similar processing pipelines; see SI: Note 1. To remove AV1451-PET scans’ considerable non-specific binding and the effect of iron in thalamus and striatum, their values were removed from subsequent analysis. The proposed network model was applied to canonical healthy connectomes, and model patterns compared against the ADNI regional data.

### Statistical Analysis and model testing

Both group statistics (t-statistics for each of EMCI, LMCI and AD groups) and individual fitting was performed. Empirical regional AV1451-PET tau was supplemented with MRI-derived regional atrophy to leverage larger sample size; atrophy is a useful surrogate for tau, with strong association^44^. The statistical test of choice is Pearson correlation strength, R, and its two-tailed p. Detailed model fitting to individual subjects is described in SI: Note 6.

We corrected each Pearson p-value with an inflation factor of 50 – the number of unique comparisons. We also performed extensive permutation tests as noted in earlier with 500 random permutations. A comparison of the reported R with “null” distributions of randomized R gives a pertinent and additional measure of significance. We further obtained Fisher’s transform and its p-values to assess significant improvements between models. AIC scores were also computed to assess whether different models with varying complexity give comparable performance.

#### Data and code Availability

All patient data used in this study were obtained from the public ADNI study. Users interested in obtaining these data can do so by directly visiting the website http://adni.loni.usc.edu. Nonetheless, for the purpose of convenience and to facilitate experimenting with the presented approach, group regional SUVr, atrophy and metabolism, etc are being made available within the code repository, and can be found at https://github.com/Raj-Lab-UCSF/Aggregation-Network-Diffusion. There are no restrictions on the use of this data. All computer code and group statistics used in producing the current results will be shared publicly and without limitations at our laboratory's GitHub site: https://github.com/Raj-Lab-UCSF/Aggregation-Network-Diffusion. There are no restrictions on the use of this code, subject to standard BSD Clause-3 license terms.

## RESULTS

### I. Cross-sectional relationships between tau, amyloid and atrophy

**Figure 2.**
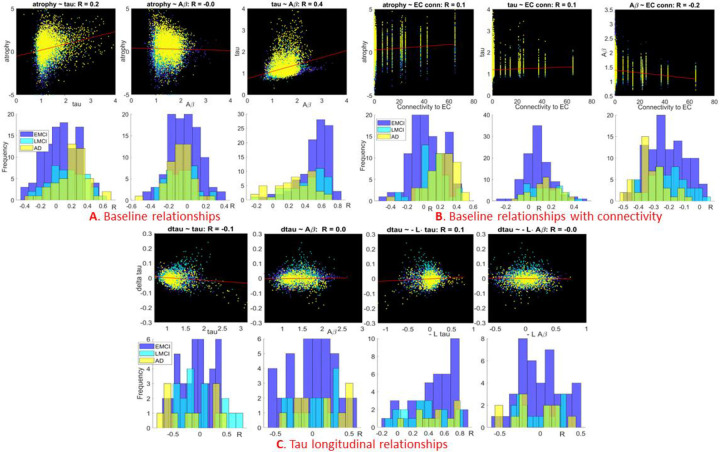
Cross-sectional and longitudinal relationships between tau, amyloid, atrophy and the network. **2A**: Scatter plots (top) and histograms of correlation strengths (bottom) are shown. There is a moderate yet significant relationship between atrophy and tau and amyloid and tau, but not between atrophy and amyloid. There is a distinct stage-dependent effect whereby later stages give stronger associations with respect to tau but the opposite for amyloid. **2B**: Using bilateral ERC as the canonical pathology seeding site, it was found that its network connectivity to a region is moderately associated with atrophy and tau but negatively associated with amyloid. Accompanying histograms suggest a stage dependency, with earlier stages giving a stronger association with connectivity for atrophy and tau, and the reverse for amyloid. **2C**: change of tau from baseline to year 1 is associated moderately with baseline tau but not baseline amyloid. To assess the hypothesis that baseline pathology may affect change of tau in a distant but connected region we employed the simple association proposed by the well-known network diffusion model: $\frac{d\tau }{dt}\propto -L\tau$, where L is the network Laplacian matrix (see model and cite ??). This remote-association was significant for baseline tau but not for amyloid, and again there was a stage-dependent behavior for tau.

The public ADNI3 study^33^ was processed with home-grown software pipelines to obtain reginal values of the three imaging biomarkers: atrophy. Tau and amyloid; see demographic data in Table S1. First we ascertained what the ADNI data at large are telling us about how the three imaging biomarkers are related to each other, and to the network. In Figure 2 we depict these relationships across the whole ADNI database of how induvial subjects triplets (tau, atrophy, amyloid) are related. Figure 2A show the three biomarkers’ associations –in the scatter plots in top row each dot represents a single subject’s single brain region. At this aggregate level clearly there is a moderate yet significant relationship between atrophy and tau, but not between atrophy and amyloid. There is a strong relationship between tau and amyloid – cementing the prior expectations that amyloid and tau are colocalized. The bottom row shows histograms of the correlation strengths of the three disease groups (EMCI, LMCI, AD) separately. It demonstrates a prominent stage-dependent effect, whereby atrophy is more tightly related to tau in later than earlier stages. The situation is reversed for the tau-amyloid associations: earlier stages have a stronger association than later stages. These data point to the well-known finding that amyloid plays a role early in AD pathophysiology, and at later stages it has a plateauing behavior and is no longer predictive.

#### Stage-dependent relationship to network connectivity

Figure 2B shows analogous results, but with the x-axis depicting the network connectivity strength of each region to the canonical seed region of bilateral ERC. This data attempts to test at the population level the hypothesis that biomarkers are predicted by connectivity to the pathology origination site. At the aggregate level we find moderate but significant evidence for an association between EC-connectivity and all three biomarkers; however the association with amyloid is in fact negative. These results are also stage-dependent; with earlier stages giving a stronger association with connectivity for atrophy and tau, and the reverse for amyloid.

#### Longitudinal relationships between tau, amyloid and network connectivity

The longitudinal behavior of tau was assessed using the first difference between baseline and year-1 visit, the most generalizable longitudinal measure for subjects with varying number of visits. Figure 2C shows these relationships at the aggregate level (top) and for each stage (bottom). A moderate but significant association with change of tau was found for baseline tau, but not baseline amyloid. We then generalized the network hypothesis to assess whether baseline pattern of tau weighted by each region’s network connectivity would predict the change of tau. This hypothesis involves the possibility of remote effect, whereby tau at a region may after the change of tau at a distant but connected region. We used a simple model of spread of tau from baseline pattern along the network, given by the well-known network diffusion model (Raj 2012). As shown in the figure, this hypothesis was found to have significant support for baseline tau, but not for amyloid. As with earlier results, there is a distinct stage dependency, such that earlier stages show a stronger association for baseline tau but not for amyloid.

Taken together, these results demonstrate that there is a significant cross-sectional and longitudinal relationship between tau and amyloid distributions in the Alzheimer brain, and this relationship is stage-dependent.

### II. Evidence-based development and adjudication of competing hypotheses of protein aggregation, network transmission and interaction

The above data snapshots provide empirical support to the hypothesis of a cross-species interaction between Aβ and tau; however it does not point to the exact mechanistic form these interactions might take, nor does it give a model of brain-wide protein propagation. Whether these data are consistent with connectome-mediated spread, or with other modes f spread, e.g. based on proximity or along fiber distances, is also unclear. In this section we will first develop a base model of network transmission of tau and Aβ. From the base model we will create various extensions that will implement different potential interactions, as listed in the Model section of Methods. Table 1 summarizes these 7 competing hypotheses, and the computational model used to assess and adjudicate each hypothesis quantitatively.

#### Illustration of base model of joint evolution of Aβ and tau

**Figure 3.**
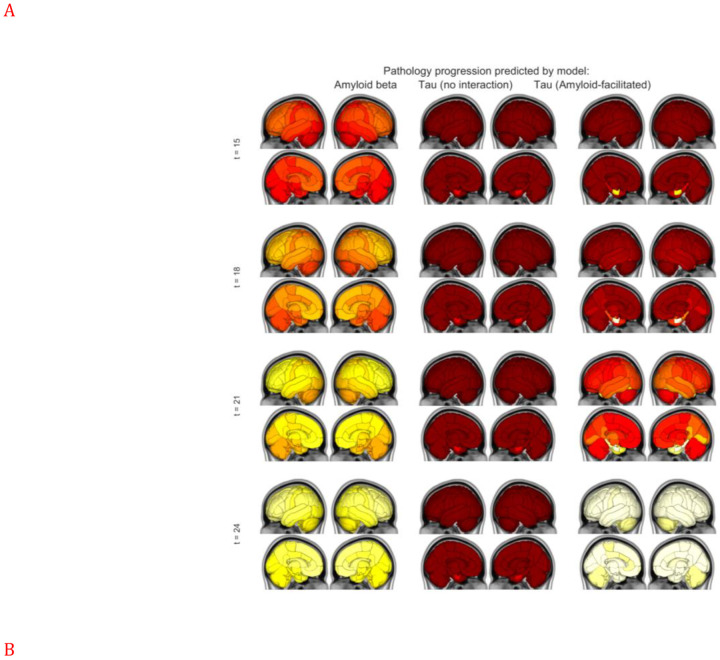

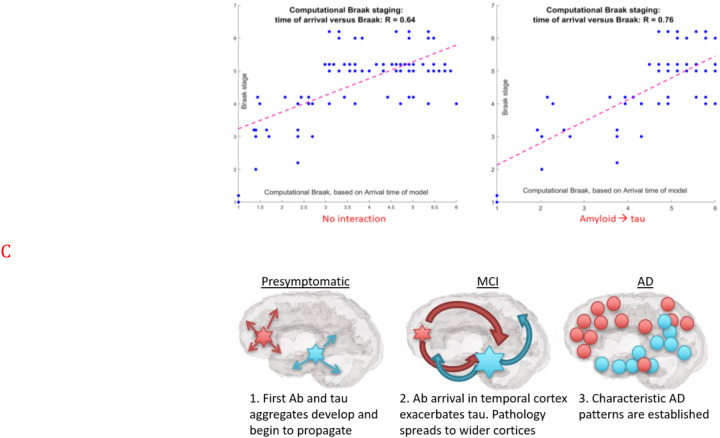
The evolution of model dynamics of amyloid and EC-seeded tau. **A**: **Spatiotemporal evolution** of the model evaluated by numerical integration of Eqs (1a, 2a), on the 86×86 healthy Desikan connectome using default model parameters. The first column shows the evolution of amyloid, the second column of amyloid-facilitated tau and the third column of tau only, putatively a model of primary age-related tauopathy (PART), which remains confined to the temporal cortex and is relatively lower in magnitude compared to AD. In comparison, the amyloid-facilitated tauopathy model (middle column) shows much more profuse tau deposition in temporal areas with the arrival of amyloid into temporal cortices, putatively recapitulating AD tau. Each sphere represents a brain region, its diameter is proportional to pathology burden at the region, and is color coded by lobe (blue = frontal, purple = parietal, red = occipital, green = temporal, cyan = cingulate, black = subcortical). **B**: **Predicting Braak stages using computational model.** Using the time-of-arrival calculation of each group’s fitted eNDM model, we developed a 6-stage “computational Braak” staging system. The predicted Braak stages strongly agree with the original Braak stages, applied to the DK atlas parcellation, achieving *R* = 0.76, *p* < 10^−6^ for the best-adjudicated model (right). In comparison, the non-interacting model (left), while being significant at *R* = 0.64, is substantially worse, suggesting that amyloid→ tau interaction is a necessary factor in Braak staging. **C**: **Gestalt of spatiotemporal progression of AD-related amyloid and tau**. Following diffuse production of amyloid **(red)** in proportion to metabolism, and focal production of tau **(blue)** at the EC, aggregation into plaques and tangles occur at networked sites following graph topology. Tau pathology is further aggravated by the arrival of amyloid in temporal areas. Finally the classic AD spatial patterns are established – frontal-dominant for amyloid and temporal dominant for tau.

The numerical solution (**x**_**Aβ**_(*t*), **x**_**τ**_(*t*)) of the joint Aβ-tau model (Eqs 1a,b) was evaluated on the canonical healthy connectome under the Desikan-Killiany parcellation (86 regions). In order to assess the model behavior broadly, in the following set of results we used a canonical model specification, given by the coarsely optimized (default) parameter values. Please refer to SI: Note 6 and Table S2 for details. Figure 3 left column shows the evolution of amyloid regional distribution, as it propagates into the structural network, eventually establishing itself in frontal and medial areas of the brain. Model amyloid evolution appears to recapitulate the classic amyloid progression, e.g. Thal’s five pathologic stages^2^ and amyloid PET patterns^3^; proceeding from medial frontal and precuneus, areas with high baseline metabolism, into the wider network, only slowly entering temporal cortices. The middle column shows the evolution of tau on the same network, starting from seeding event in the bilateral ERC.

#### Network transmission of Aβ and tau, with cross-species interaction

Each of the 7 interaction models, encompassing the most common mechanistic hypotheses in the field, were implemented as described in Model section. For illustration purposes here we describe the most promising of those, given by the 1-way interaction from amyloid to tau; specifically: **Aβ-facilitated tau aggregation**^16–18^ encompassed in (Eq 2). The resulting tau progression, illustrated in Figure 3A right column, remained confined to the medial temporal lobe until sufficient levels of Aβ had spread to and accumulated there (at *t* ≈ 10). There onward, in contrast to the “pure tau” evolution, it took on a more aggressive trajectory, spreading first to nearby limbic, then basal forebrain, then parietal, lateral occipital and other neocortical areas, in close concordance with Braak’s six tau stages^1^. Thus, the facilitation of tau by amyloid does not lead to colocalization of the two until late stages, helping explain why regional Aβ patterns do not coincide with tau and atrophy patterns. Conversely, the absence of the interaction term led modeled tau to remain confined to the temporal lobe (Figure 3A, middle), mirroring primary age-related tauopathy (PART), a new classification for mild neurofibrillary degeneration in the medial temporal lobe, but no Aβ plaques^34^. For comparison, the AAL-connectome-based model evolution is shown in Figure S4, with very similar behavior, indicating that the choice of atlas or processing pipeline did not drive results.

Supplemental Figure S3 shows the global accumulation of theoretical pathology over model time, evaluated at default parameters. All proteins increase over time, but amyloid-facilitated tau diverges dramatically from the non-facilitated tau at around t=15, mirroring Figure 3. Figure S3 makes it clear that while the “pure” tau model also captures empirical data, it does so far slower and achieves far less correlation strength than the facilitated version.

##### Predicting Braak stages using computational model.

Using the time-of-arrival calculation of each group’s fitted eNDM model, we developed a 6-stage “computational Braak” staging system. The predicted Braak stages strongly agree with the original Braak stages, applied to the DK atlas parcellation, achieving *R* = 0.76, *p* < 10^−6^ for the best-adjudicated model (right). In comparison, the non-interacting model (left), while being significant at *R* = 0.64, is substantially worse, suggesting that amyloid→ tau interaction is a necessary factor in Braak staging.

**To summarize** these impressions, we find that **amyloid**→**tau** interaction is an essential component of the spatiotemporal propagation of the two species, without which the computational model does not fully recapitulate empirical amyloid and tau spatial patterns in patients. An overall picture that emerges can be depicted (Figure 3C) whereby diffuse production of amyloid, potentially in proportion to metabolism, and focal production of tau at the EC, propagate into the network, even as tau pathology is further aggravated by the arrival of amyloid in temporal areas.

#### Group level empirical validation and model fitting

**Figure 4.**
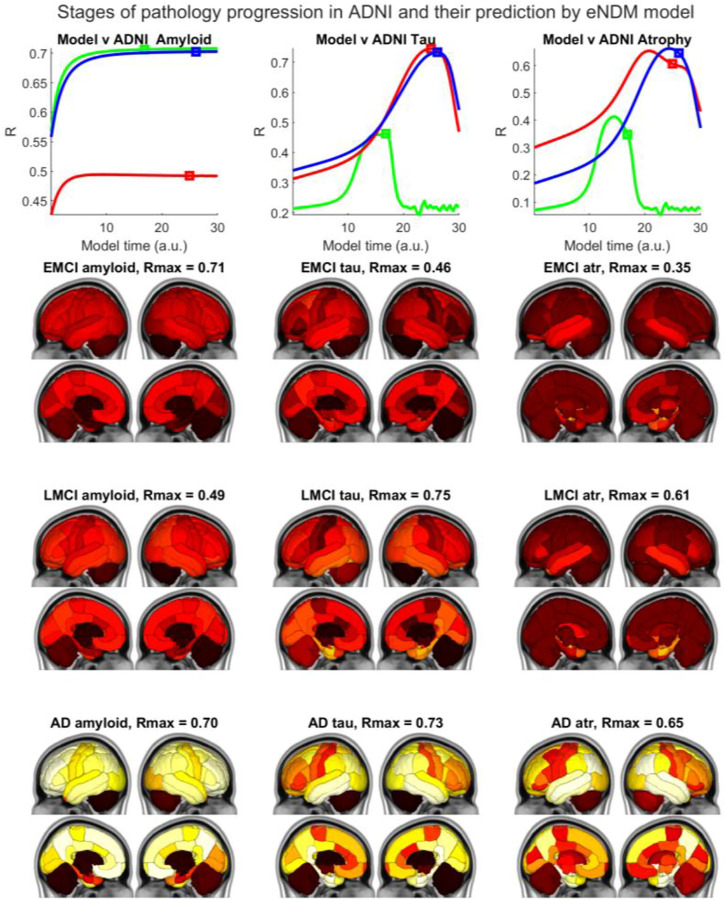
Validating evolution of model tau seeded at ERC against empirical data. **Left column**: The top panel shows the behavior of model evidence R against model time t, suggesting that the model reaches a peak correspondence with empirical data relatively early, then plateaus. The model time *t*_*max*_ at which the model was evaluated against empirical data is denoted by square markers. The “glass brain” renderings show empirical amyloid-PET SUVr patterns from all 3 diagnostic groups. The R statistic between the model and empirical data are shown alongside. **Middle column:** The top panel shows that the R between the model and empirical tau data grows steadily, hits a peak, then declines. Notice that the peak for tau occurs more than a decade (in model “years”) after the plateau seen for amyloid. R is strongest for LMCI and AD and weakest for EMCI. The cross-sectional empirical tau-PET SUVr patterns from all 3 diagnostic groups are shown. The **right-most column** shows results for MRI-derived atrophy. The best-fitting model amyloid and tau patterns appear strikingly similar to the empirical PET data, which a quick visual comparison of Figures 3 and 4 will confirm. The match between the model and empirical data are strong for LMCI and AD, and weakest for EMCI. Note that *t*_*max*_ for each case is identical between amyloid, tau and atrophy – this point may not always coincide with the actual peak R for a given case.

We next performed model fitting on empirical group data of the proposed model initiated at the canonical EC-seeding of tau. For model fitting we developed a robust maximum a posteriori (MAP) inference procedure described in detail in SI: Note 6. Cross-sectional group ADNI data were correlated against the fitted model’s evolution (**x**_**Aβ**_(*t*), **x**_**τ**_(*t*)) at every time *t*, and Pearson’s R was recorded. The resulting “R-t curves”, shown in Figure 4 displayed a characteristic peak as more amyloid and tau pathology diffused into the network it increasingly recapitulated cross sectional data. Subsequently model diverged from empirical pattern, decreasing R. Since our model posits that both amyloid and tau evolution happens on the same time axis, we report the model instant that maximized the posterior, rather than peak R for either tau or amyloid separately. The R value resulting from this “shared time”, called *t*_*max*_, is recorded for both amyloid and tau (denoted by square markers) and considered as model evidence. Optimal fitted parameters shown in Table S2 indicate substantial differences between groups. Table 2 tabulates the resulting R statistics. All reported R values that are moderately to highly significant as denoted by * (p < 0.05) and ** (p < 0.005), **corrected for multiple comparisons.** Statistics for all model forms are contained in Table 2; however for illustration purposes below we highlight the best, most parsimonious interaction model (1-way amyloid → tau interaction).

##### Amyloid.

Correlations between model and empirical AV45 SUVr are shown in Figure 4 and Table 2. The peak R was: *R* = 0.71 (EMCI), *R* = 0.49 (LMCI) and *R* = 0.70 (AD). All correlations were highly significant. R-t curves shown suggest that the correspondence reaches a peak and then plateaus. Note that the model time t has arbitrary units that may not be directly comparable to empirical duration in years. **Tau.** The network propagation of modeled tau starting from its seeding in EC was computed and the correspondence between model-predicted regional tau deposition and group-average empirical AV1451-PET data, was assessed (Figure 4, Table 2). Unlike the amyloid case, the R-t curves of tau and atrophy show a slow and steady rise and peak without a plateau effect. The R between the model at *t* = *t*_*max*_, and empirical tau PET data, was: *R* = 0.51 (EMCI), *R* = 0.75 (LMCI) and *R* = 0.73 (AD). The latter two correlations were extremely highly significant. Striatal AV1451 uptake is considered non-specific binding and was suppressed in these comparisons. Correlation with EMCI group is poor, likely due to lower PET uptake and inter-subject heterogeneity. **Atrophy**. Since tau is highly co-localized with regional atrophy, we also show a comparison with MRI-derived group atrophy. However, we did not fit the model again to atrophy, instead borrowing the tau-fitted models for each cohort, under the assumption that tau is the primary, and atrophy is a surrogate for tau. The resulting R between the model at *t* = *t*_*max*_ and empirical atrophy was: *R* = 0.37 (EMCI), *R* = 0.60 (LMCI) and *R* = 0.66 (AD). EMCI atrophy gives the lowest correlation, likely due to the low effect size measurable on MRI in this cohort.

##### Time of peak:

The *t*_*max*_ of maximum posterior followed the expected order: *t*_*max*_ for AD > LMCI>EMCI. It is challenging to fit an accurate time axis to empirical data, since it does not have a measure of pathology duration – which may never be known. This order also supports the ability of the fitted time of arrival as an accurate Braak staging of the disease (see above). Interestingly, the peak for tau occurs more than a decade (in model “years”) after the plateau seen for amyloid – perhaps recapitulating well-known clinical and pathological examinations that suggest a decade-long delay between the two processes.

#### Quantitative adjudication of different models of amyloid-tau interaction

We evaluated five theoretical interaction models: **1)** No-interaction model Eqn (1); **2)** 1-way interaction model (Eq 2), whereby tau affects amyloid but not vice versa; **3)** 1-way interaction whereby amyloid affects tau aggregation but not vice versa (Eqs 3); **4)** 1-way interaction whereby amyloid affects tau diffusion into the network, but not vice versa (Eqs 4); and **5)** 2-way interaction model (Eq **5**). Each model was evaluated in identical fashion after full MAP inference and identification of a single unique operating time *t*_*max*_ that spans both amyloid and tau data. Statistical comparison of these fitted models is shown in Table 2. Both the fitted Pearson’s R is reported, along with model comparison using Fisher’s R-to-z transform and the p-value of significant differences between models; p<0.05 are highlighted.

These are our findings: The best overall model is the 1-way interaction ***Aβ*** → **tau aggregation**. In contrast, the 1-way interaction *Aβ*→ tau diffusion does not significantly improve upon the base (No-interaction) model, suggesting that amyloid primarily facilitates the aggregation of tau, not its enhanced spread into the network. The 2-way model is insignificantly different from the 1-way model. Both interaction models are significantly better than the no-interaction model. Interestingly, 1-way tau→*Aβ* model is indistinguishable from No-interaction model, suggesting that tau does not exert a clinically relevant effect on amyloid.

To confirm these impressions and to rule out improved performance due to higher degrees of freedom of additional coupling coefficients, we obtained the Aikeke information criterion (AIC) for each model. Model order was defined as the number of non-zero parameters. The lowest AIC is always achieved by 1-way *Aβ* → tau interaction model, supporting our earlier conclusion. AIC for *Aβ* → tau interaction is always higher than for tau→*Aβ* or 2-way interaction, confirming that additional tau→amyloid interaction does not further improve model fit. The five models are ordered descendingly in AIC:

**(Best)** *Aβ*→tau aggregation (local) **>** *Aβ*→tau aggregation (remote) **>** tau←→*Aβ* **>** *Aβ*→tau diffusion **>** no-interaction > tau→*Aβ* aggregation **(Worst).**

The amyloid component is equally strong for all models and its parameters are largely unaltered – this might reflect the well known plateau effect of amyloid, although further investigation will be warranted.

#### Permutation testing to demonstrate disease specificity

Various permutation tests were deployed to determine whether the presented model only recapitulates empirical regional distributions when it is applied in the correct region order and to the correct human connectome, contained in SI: Notes 7 and Figures S5 and S6. Under 500 random permutations of atrophy and tau (Figure S5-A), and under 500 random permutations of the connectome itself (Figure S5-B) we found that the above-reported R values of the true data are highly significant compared to these “null” distributions (*p* < 10^−3^ for all groups). This was true whether for Pearson correlation or Spearman (Figure S6) as the performance metric.

#### Comparison of different modes of spread

We implemented two alternative modes of spread: a) inter-regional pathology spread depends only on the shortest Euclidean distance; and b) transmission between regions is inversely proportional to the average length of fiber projections between regions. See Methods and quantitative comparison in Table 3. Again, the model in each case was refitted using the MAP estimator, hence each fitted model may represent the best-case scenario for that hypothesis. P-value of Fisher’s R-to-z transform of significant differences between models was evaluated, accounting for the correlation between dependent models. We found that : **a)** For both tau and amyloid evolution, connectome-mediated spread model gave the closest correspondence to empirical data; and **b)** Euclidean spatial spread was slightly but not significantly superior to fiber distance-based spread. For these comparisons we chose the best interaction model identified in Table 1 (*Aβ* → *tau* aggregation) for all three networks. However, we repeated these comparisons using other interaction terms as well, and did not find significant differences in performance (Fisher’s p-value = N.S.).

#### Translational aspects

Having established the network model’s validity on group data, and having determined which modes of the tau-amyloid-network interactions are relevant and empirically supported, we devote the last section to showcase two key results related to translational aspects in patients: etiologic heterogeneity and the capacity to predict an individual subject’s spatiotemporal trajectory of AD. These results will serve as the foundation of future diagnostic and prognostic applications.

##### A) Uncovering etiologic heterogeneity: Repeated seeding to assess alternative seeding sites

**Figure 5.**
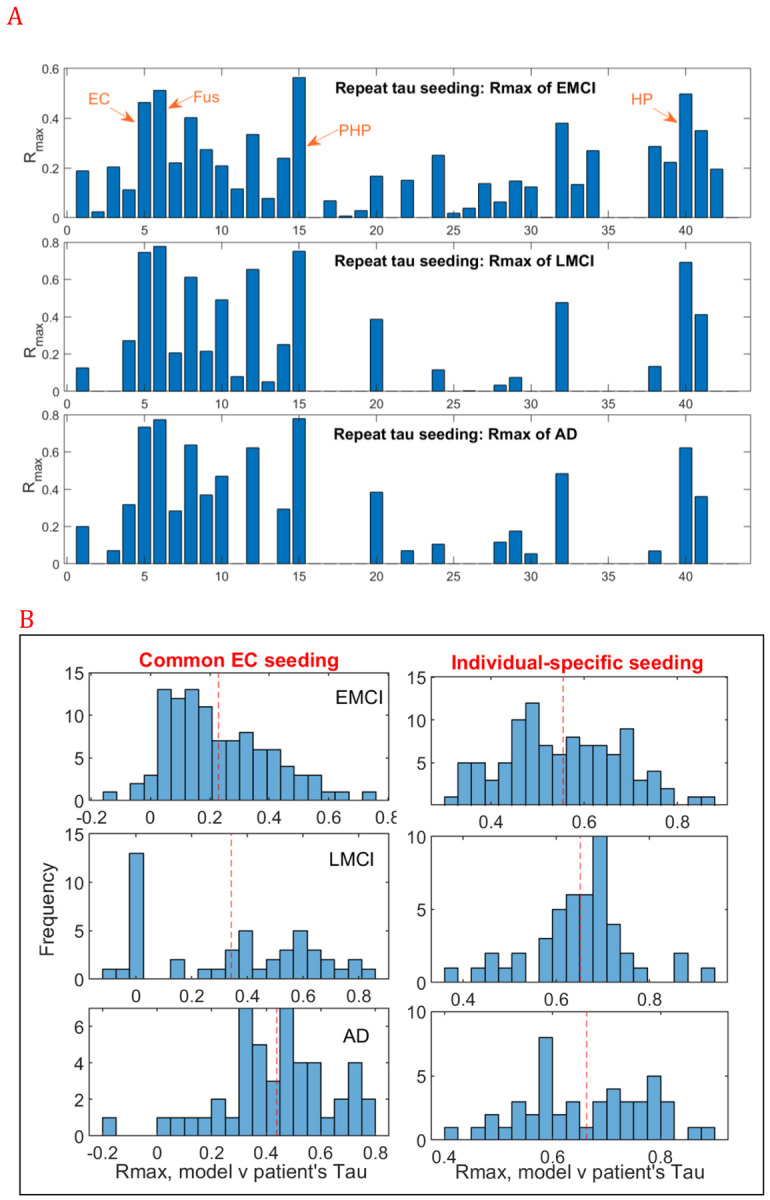
Translational applications. **A:** **Bar charts of repeated seeding of each region in turn**. For each seed region, peak Pearson’s R over model time, corresponding to the correlation between the best interaction model (amyloid-facilitated tau aggregation) and empirical tau PET data, is shown, for each of the 3 diagnostic groups: EMCI (top), LMCI (middle) and AD (bottom). Entorhinal cortex (EC) is amongst the best cortical seeding sites and Hippocampus (HP) the best subcortical site. Other likely seeding sites include Fusiform (Fus) and Parahippocampal gyrus (PHP). **B**: **Fitting model to individual subjects.** Histogram of Rmax achieved by fitting the model parameters to individual subjects’ baseline regional tau data, after imposing a common seeding site located at the ERC (Left) or after using the best seeding site for each subject (Middle). Amyloid histograms are shown on Right panel. Here the best model combination from Table 2 was used: 1-way interaction (*Aβ* → *tau*) on the structural connectivity graph, and the evaluation was performed on tau-PET scans in ADNI3. Average Rmax over all subjects within each diagnostic group is shown in red dashed line.

Entorhinal cortex (EC) was chosen above as the canonical tau seeding site. To establish other regions’ other regions’ seeding plausibility we repeatedly simulated the selected network interaction model seeded from every possible region bilaterally. For each seed region, peak Pearson’s R between model and ADNI tau PET data is shown in Figure 5A. EC is amongst the best overall cortical seeding site, while hippocampus (HP) is the best subcortical site. Other prominent seeding sites include Parahippocampal gyrus (PHP) and Fusiform gyrus (Fus), which are adjoining EC and appear frequently similar in tau uptake to EC on PET imaging. Thus the quantification of these structures as likely seeding locations affirms our model’s relevance and plausibility. The evolution of pathology from some of these non-EC sites are illustrated in Figures S8 and S9, for HP-seeding and IT-seeding, respectively. They remain substantially similar to Figure 3; however a notable difference from EC seeding is that HP seeding leads to higher involvement of medial temporal and subcortical structures. Amongst the above 5 regions, EC is unique in giving consistently one of the highest seeding likelihood despite having less levels of empirical tau deposition than others.

##### B) Individual subject fitting and prediction

For translational applications we must necessarily move away from above group comparisons to model inference on individual subjects, while accommodating both etiologic heterogeneity as well as potentially subject-specific model parametrization. In order to achieve this we deployed our robust the maximum a posteriori (MAP) inference procedure (see SI: Note 6) on individual subjects’ multimodal regional biomarker data from ADNI-3 (see Table S1 for demographic details), under our best-adjudicated model (i.e. network-mediated spread of tau and the 1-way interaction best *Aβ* → *tau* aggregation). Figure 5B gives the histograms of Pearson correlation *R*_*max*_ between observed and model-predicted regional tau distribution in each subject. For comparison we show the results of both canonical (entorhinal) seeding and each individual’s best seeding site. EC seeding was significantly worse (paired t-test after Fisher R-to-z: *p* < 10^−7^, corrected), implying that a common seeding site may not be appropriate for all subjects, and revealing a potential etiological factor behind observed heterogeneity in AD-spectrum subjects. With individual-specific seeding and model fitting, we were able to achieve excellent prediction of the subjects’ tau distributions, with Rmax ranging widely up to a maximum of 0.9 and mean of 0.65 for LMCI and AD, and slightly lower for EMCI subjects. The mean Rmax across individuals in Figure 5B roughly tracks, but slightly lower than, the Rmax achieved on the group-averaged tau data of Figure 4 and Table 2; which is expected due to heterogeneity as well as measurement noise in individual subjects. It is also noteworthy that canonical EC seeding fails in approximately half of the subjects in eMCI and LMCI cohorts (*e*. 𝑔. *R*_*max*_ < 0.3) but only a small minority of diagnosed AD patients: indicating that higher stages have lower etiologic heterogeneity.

## DISCUSSION

The protein-protein interaction of amyloid and tau in neurodegenerative diseases is a central feature and key to understanding AD pathophysiology^17,30–32^, now commonly called the A-T-N model^38^. However, this model has been difficult to reconcile with the observations of dissociated spatial distribution of tau and amyloid. Critical aspects of brain-wide propagation of tau and amyloid, and the exact mechanistic form their mutual interactions, remain unsupported empirically in human disease. Hence the cause-effect mechanisms by which amyloid and tau regulate each other and cause downstream neurodegeneration and symptomatology, remain poorly understood. Prior mechanistic studies in model organisms reveal a plethora of potential interactions, yet these possibilities are difficult to test directly in humans. This is problematic since animal models do not fully recapitulate human disease. Further, data from experimental systems are sensitive to idiosyncratic methodological choices, leading to a diversity of mechanistic possibilities that may not generalize and may not be germane to human disease. Yet, the interrogation of these aspects is critical to achieve disease understanding and future therapeutic options.

In this study we took a computational rather than experimental approach to address these issues directly in human AD. Our objectives were twofold: First, to test whether a mathematical encoding of network spread due to trans-neuronal proteopathic transmission^19,23,39^, combined with local interaction between amyloid and tau pathologies, is capable of recapitulating observed pathology progression in AD; Second, to infer quantitatively the factors of cross-species interactions, their causal direction, and their associated kinetic rate parameters in human AD brain.

Our major findings are: First, the local production of Aβ driven by glucose metabolism followed by subsequent network spread correctly recapitulates the spatial distribution of empirical Aβ, especially in the frontal lobe, the region of highest glucose metabolism. Second, starting from EC, model tau recapitulates empirical tau in temporal areas, followed by network ramification in wider cortices. Third, introduction of Aβ→tau interaction in the model recapitulated the concept of amyloid-facilitated tauopathy, and was critical for success in recapitulating empirical data. Fourth, 2-way interaction is equal or worse than 1-way mode in recapitulating empirical patterns, and the reverse interaction (tau→Aβ) gives the poorest model evidence. Fifth, connectome-mediated spread outperforms other modes of spread, whether by proximity or by fiber length. Sixth, we verified these group-average results on individual subjects, both at baseline and longitudinally, and found essentially equivalent results. These results support an amyloid-facilitation rather than the classic amyloid-cascade hypothesis, such that both amyloid and tau are capable of independently ramifying within the network, but with an interaction between the two. As discussed below, these results help explain certain known but poorly understood aspects of AD.

### Network transmission drives divergent spatiotemporal progression of Aβ and tau

We find that Aβ production driven by glucose metabolism and local APP pool, followed by a certain amount of network dissemination, recapitulates regional pattern of empirical amyloid deposition (see early peak in the R-t curves of Figure 4). Neural activity is known to regulate the production and secretion of Aβ^3^. In young transgenic mice, neural activity is related to Aβ release and later deposition of plaques^25^. Neural activity modulates the release of cleavage products of the amyloid precursor protein^40^, and Aβ secretion could be affected by neural activity^41^ through synaptic exocytosis^26^. In humans, Aβ release parallels fluctuations in synaptic activity in human sleep/wake cycles^42^. AV45 uptake is higher in hubs, multimodal cortices^43^ and default mode network, all characterized by higher baseline metabolism^27^. These data provide strong molecular and imaging basis of the amyloid production model (Eq 1a).

For the simulations that were explicitly seeded at EC, their ability to correctly identify mesial temporal involvement is not surprising. However, model pathology increased (Figure 3) and fit improved with model time (see R-t curves), suggesting that network spread into other areas was essential to improve the model’s performance. **Repeated seeding** shown in Figure 5A confirms that EC and adjacent temporal areas are excellent tau seeding locations. Given the preponderance of prior evidence, this serves to further enhance the plausibility of the proposed model.

Once Aβ and tau pathologies are anchored at their respective early sites, subsequent network transmission via the non-linear ODE model of Eqns (1,2) correctly recapitulated the spatiotemporal progression of both Aβ and tau. Thal’s five pathologic stages^2^ and amyloid PET patterns ^3,35^ were correctly recapitulated by the model’s Aβ evolution, proceeding from medial frontal and precuneus, areas with high baseline metabolism, into the wider network, only slowly entering temporal cortices. These results correlated significantly with amyloid PET scans of disease cohorts. In contrast to Aβ, model tau proceeded outward from EC, to lateroinferior temporal cortices, thence to parietal, lateral occipital and medial frontal areas, in close concordance with Braak’s six tau stages^36^. The R-t curve of the tau model against AV1451-PET data (Figure 4) yielded highly significant *R* = 0.75 (LMCI), *R* = 0.73 (AD). Against group amyloid, the model yielded very high significance: *R* = 0.71 (EMCI), *R* = 0.70 (AD) Further, the model correlated with MRI-derived regional atrophy of EMCI, LMCI and AD cohorts with high significance. MRI atrophy is an excellent surrogate for underlying tau, as known from post-mortem studies^44,45^. The presented correlations are significantly stronger than 500 random permutations (Figure S5), suggesting that the model is specific to the human connectome and brain topography.

That the spatiotemporal patterning of *Aβ*, tau and atrophy are distinct is well-known but not fully understood^4^ and appears at odds with the prevailing amyloid cascade hypothesis^3,46^. Soluble Aβ is sometimes invoked to help explain the discrepancy, but this has been questioned^3^. The divergent evolution of theoretical amyloid and tau on the same network suggests that network spread combined with metabolic drivers might be the governing mechanism behind these long-observed discrepancies. Importantly, this spatial divergence holds even in the presence of amyloid-facilitation.

### Aβ-facilitated tau ramification

Large-scale connectivity approaches have yet to address the role of protein-specific mechanisms in disease evolution^47^. Generic networked spread, e.g. our previous study^23^, cannot capture the divergence between amyloid and tau, which here was made possible by the introduction of new interaction terms. Our third major result is that Aβ-tau interaction played an important role in forcing the extra-temporal spread of tau. The presence of Aβ is known to influence the course and severity of tau and atrophy^16–18^. Ittner and Götz^17^ suggested three modes of interaction: (1) Aβ drives tau pathology; (2) synergistic toxic effects of Aβ and tau; and (3) tau may mediate Aβ toxicity. Aβ and pathological tau co-localize in synapses^48,49^, and tau is essential for Aβ-induced neurotoxicity^50^. Aβ seeded exogenously in tau transgenic mice elicited aggressive tau pathology in retrogradely connected regions^18^. Amyloid pathology accelerated tau deposition in double transgenic mice, but the reverse effect was not observed^16^. A “seminal cell biological event” in AD pathogenesis was suggested, whereby acute, tau-dependent loss of microtubule integrity is caused by exposure of neurons to readily diffusible Aβ^32^. However some studies have not found an explicit amyloid-mediated increase in tau in humans. It was suggested that tau propagation across connected regions is unlikely to be a gain of function mediated by the presence of amyloid-β^51^. Nonetheless, there are other mechanistic routes to enact this interaction, e.g. Aβ-related microglial activation promotes local tau-hyperphosphorylation^52^, increasing the likelihood of tau spread across connected neurons^53^. Microglia PET in AD confirmed early microglial activation in inferior temporal sites of tau accumulation^54^. Hence it is possible that instead of a direct interaction between amyloid and tau, there may be an indirect mediation via microglial activation, tau hyperphosphorylation, increased tau load and subsequent enhanced network spread of tau. An excellent review of tau-amyloid synergy is available^55^.

In our mathematical exposition of these aspects, modeled tau seeded at EC remained confined to the temporal lobe until sufficient levels of Aβ had spread to and accumulated in temporal cortices (Figure 3). Thereupon amyloid-facilitated model tau started diverging from pure tau, taking on a more aggressive trajectory, spreading first to nearby limbic, then basal forebrain, then other neocortical areas. Thus, the facilitation of tau by amyloid does not lead to colocalization of the two until late stages, further helping to explain why regional Aβ patterns do not coincide with tau and atrophy patterns^55,57^. These results concord with neuroimaging, e.g. Sepulcre et al found several convergence zones where amyloid and tau might undergo potential interactions, especially in inferior–lateral temporal areas and entorhinal cortex^58^, which they link to the high density of tau in dystrophic neurites in the inferior–lateral and posterior temporal areas^59^. Using a local-to-distributed approach they find specifically that tau accumulation in these temporal areas relates to “massive Aβ elsewhere in the brain” (see, e.g. Fig 6 therein), suggesting these areas as critical regions for linking both pathologies at the large-scale level. Franzmeier 2019 show that tau uptake in amyloid-negative healthy elderly is restricted to (inferior lateral) temporal cortex, but is observed in extra-temporal areas in amyloid-positive controls, MCI and AD, in increasing order^60^ (Figure 2 therein). Remarkably, our theoretical modeling also gives the same regions (inferior temporal and EC – see Figure 3) as key areas of convergence between amyloid and tau, where the “arrival” of the former is accompanied by prominent deposition of the latter.

Conversely, the absence of the interaction term led modeled tau to remain confined to the temporal lobe (Figures 3), mirroring primary age-related tauopathy (PART), a new classification for mild neurofibrillary degeneration in the medial temporal lobe, but no Aβ plaques^34^. Thus, medial temporal NFTs may be involved in two divergent processes: AD and PART. Purely tau-specific abnormalities, e.g. the MAPT gene H1 haplotype, would predispose the subject to PART, whereas additional Aβ abnormalities, e.g. the dysregulation of presenilin, APP or APOE ε4 allele, would cause AD predisposition^34^. Our simulations provide remarkable support to this concept. The non-facilitated tau model was less successful than amyloid-facilitated tau model (Table 2, Figure 3). Overall, the pure-tau model evolved far slower (in model time) to reach peak correlation with empirical data compared to facilitated-tau model. This result might impart one of the first algorithmic evidence of the coupling between the two protein species as a relevant factor in AD spatiotemporal progression.

#### Two-way interaction model.

The interaction between tau and amyloid can take many forms; a benefit of mathematical modeling is that such interactions can be “observed” *in silico*. We evaluated all four modes of interaction between amyloid and tau. Our 4^th^ major result is that the 2-way model is insignificantly different from the 1-way model for both amyloid and tau, while they are identical for amyloid (Table 2). The tau→amyloid interaction performed relatively worse on tau data, suggesting that tau driving amyloid is not a clinically relevant factor in pathology ramification, and its addition gives poorer AIC scores. That amyloid results are identical for both modes is somewhat surprising. We think this was due to amyloid evolution peaking before a significant tau-influence was observed. Hence, by the time tau can begin to influence amyloid, the pear R against empirical amyloid distribution has already been reached.

#### Non-connectome-mediated spread.

Can modes of spread other than trans-neuronal anatomic spread also explain empirical data equally well? Our 5^th^ major result is that alternative proximity- and fiber distance-based spread models did not give good correspondence with empirical tau, and were essentially identical for amyloid (Table 3). The amyloid result mirrors our previous exploration in mouse data^37^, where too connectivity was not better than spatial spread. The tau result potentially lends a new mechanistic insight about the protein-protein interaction in AD.

### A parsimonious explanation of spatiotemporal progression of coupled pathologies in AD

The overall picture that emerges was distilled in Figure 3C. Both amyloid and tau are subject to identical networked spread processes. Amyloid production is driven by APP and metabolism, while tau is seeded in mesial temporal cortex, e.g. the EC; but otherwise both pathologies can be diffusely propagated, with no specific role for selective vulnerability due to cell type or cytoarchitecture. From the first temporal tau aggregates, network transmission continues and when sufficient amyloid has arrived at temporal cortices, tau pathology is aggravated. Finally, classic, spatially divergent amyloid and tau patterns are established. This model can explain many empirical discrepancies noted in the literature, yet has the benefit of parsimony, universality and consistency.

#### Broader Implications.

Scientifically, this study bolsters the hypothesis of trans-neuronal transmission by demonstrating its role in humans, an aspect that cannot be studied directly. Since trans-neuronal spread is a common feature of neurodegeneration, the proposed model may be equally applicable to co-morbid pathologies seen in other disorders like Parkinson’s, Lewy Body, frontotemporal and other dementias. Clinically, our model could provide a unique opportunity for computational tracking and prediction of individual patients, especially regarding their future progression. Although multiple AD imaging biomarkers are now routinely acquired, including MRI, FDG-, AV45- and AV1451-PET, the relationships between them are poorly understood, hampering clinical explorations. Since the model can successfully capture the relationship between various evolving biomarkers, it will enable multi-modality integration and tracking of patients’ imaging data; e.g. Figure 5, where individual subjects at baseline and longitudinally were successfully fitted to the joint amyloid-tau model. We showed that subject-specific seeding sites gave much higher accuracy than uniform common seeding of EC – revealing important inter-subject heterogeneity of etiology and ramification.

However, presented model will need to be strengthened with machine learning and further model inference. Several effective data-driven multi-modality ML methods are available, e.g. the multifactorial model^15^ or event-based models^28,29^. In contrast the current study focuses on modeling a specific process of aggregation and evolution of co-morbid pathologies. There is a role for both approaches. Successful tracking and prognostication can improve the odds of early detection, inform clinical care, provide patients visibility into the future, and serve as outcome measures in clinical trials of disease-modifying drugs.

#### Limitations and future work.

Neuroimaging software pipelines have several limitations in image resolution, noise and artifacts^23^. DTI suffers from susceptibility artifacts and poor resolution. PET has poor resolution compared to MRI, and AV45 and AV1451 tracers show significant non-specific binding. Tractography can under-estimate crossing fibers and long tracts. Small subcortical structures can present challenges in inferring connectivity. This study was not designed to achieve staging by fitting a quantitative time-axis in the model – which would ideally utilize longitudinal data and a measure of pathology duration.

## Supplementary Material

1

## Figures and Tables

**Figure 1.**
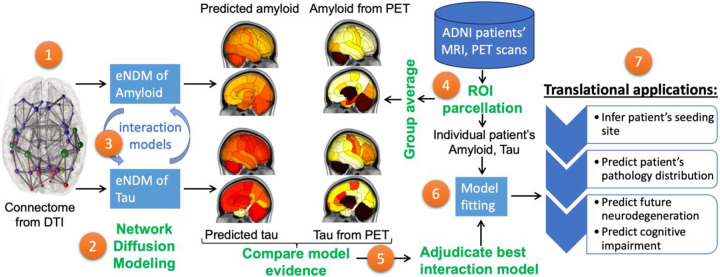
Study design, work flow and goals. Using the structural network connectome (**1**), we deploy the extended Network Diffusion Model (eNDM) that seeks to recapitulate trans-neuronal spread of amyloid and tau pathology (**2**). Startign with the base model of independent eNDMs for both amyloid and tau, we successively introduce various models of interaction between the two (**3**) – these models are listed in Table 1. From the ADNI database we extract regional distributions of tau and amyloid SUVr from raw PET images (**4**), whose group average patterns are used to statistically evaluate against each computational model (**5**). Using model evidence criteria we adjudicate between all possible interaction models, and select the best performing one, yielding a numerical assessment of the most likely mode of interaction between amyloid and tau. This adjudicated model is then fit to individual patients’ regional pathology data using Bayesian inference of model parameters (**6**). The fitted model is capable of not only predicting the patient’s pathology pattern but also their specific seeding sites – a potential marker of translational interest. The model outcomes may in future translational applications be used to predict future pathology, neurodegeneration and cognitive state of the patient (**7**).

**Table 1:** Alternative/competing hypotheses of tau-amyloid interaction to be tested in the background of transmission mediated by network connectivity.

Hypothesis	Description	Model Equation	Supporting Reference
**1) No interaction**	Tau and amyloid beta propagate independently on the connectivity network (Base model)	1a, 1b	
**2) *Aβ → tau* aggregation**	Tau and amyloid propagate on the network, but local presence of amyloid enzymatically facilitates the aggregation of tau	2a, 2b	
**3) *Aβ → tau* diffusion**	Tau and amyloid propagate on the network, but local presence of amyloid increases the spread/transmission of tau	3a, 3b	
**4) Tau *→ Aβ* aggregation**	Local presence of tau enzymatically increases the rate of aggregation of amyloid	4a, 4b	
**5) Tau ←→** *Aβ*	Tau and amyloid propagate on the network, and the local presence of each protein enzymatically facilitates the aggregation of the other protein	5a, 5b	
**6) Proximity- based transmission**	Tau and amyloid beta propagate within the brain based on proximity (Euclidean distance between regions) rather than via the connectome. They are independent of each other (i.e. under the base model 1 of no-interaction)	6a, 6b	
**7) Fiber Distance- based transmission**	Tau and amyloid beta propagate within the brain based on Fiber distance between regions rather than via the connectome. Fiber distance is the aergae length of white matter tracts that cnnect any two regions. They are independent of each other.	7a, 7b	

Brief description of each hypothesis is given, along with the mathematical differential equation corresponding to it. References to supporting literature for each hypothesis is provided. Two additional models of spread are given, pertaining to spread via proximity and spread via fiber distance – for these models we use the base case of no interaction between tau and amyloid.

**Table 2:** Peak Pearson’s R of correlation between model and empirical regional statistics.

Empirical data	No- interaction Model	1-way interaction, tau→*Aβ* aggregation	1-way interaction, *Aβ*→tau diffusion	1-way interaction, *Aβ*→tau aggregation	1-way (remote) interaction *C*⋅*Aβ*→tau aggregation	2-way interaction *Aβ*←→tau
EMCI Aβ	0.71 ** (163)	0.71 ** (163)	0.71 ** (163)	0.71 ** (163)	0.71 ** (163)	0.71 ** (166)
LMCI Aβ	0.49 * (223)	0.49 * (223)	0.49 * (223)	0.49 * (223)	0.49 * (223)	0.45 * (229)
AD Aβ	0.70 ** (333)	0.70 ** (333)	0.70 ** (333)	0.70 ** (333)	0.70 ** (333)	0.70 ** (335)

EMCI tau	0.33 (200)	0.38 (202)	0.35 (201)	**0.48** * **(190)**	**0.48** * (192)	0.36 (203)
LMCI tau	0.60 ** (232)	0.60 ** (234)	0.49 * (247)	**0.75** ** **(206)**	0.73 ** (211)	0.70 ** (219)
AD tau	0.57 ** (322)	0.51 * (332)	0.52 * (331)	**0.73** ** **(296)**	0.71 ** (301)	0.65 ** (315)
EMCI atrophy	0.34 **(171)**	0.32 (176)	0.33 (175)	0.35 (174)	0.33 (178)	**0.37** (175)
LMCI atrophy	0.60** **(158)**	0.60** (162)	0.56* (171)	0.62** **(158)**	0.61 ** (159)	0.48 * (184)
AD atrophy	0.52* (316)	0.38 (331)	0.39 (330)	0.66** **(299)**	0.62 ** (304)	0.58 ** (312)

Highly significant correlations: ** (p < 0.001), moderately significant: * (p < 0.01), after correction for multiple comparisons – i.e. inflation by n=50. Six theoretical interaction models’ evaluations are presented: the No-interaction model, without the interaction term (*γ* = 0); the 1-way interaction model, whereby tau affects amyloid but not vice versa; another 1-way model whereby amyloid affects tau but not vice versa; and the 2-way interaction model where both tau and amyloid affect each other via *γ*. Three different modes of causal interaction from amyloid to tau are given: where amyloid influences the diffusion of tau; where it influences only the aggregation of tau; and where amyloid influences tau aggregation remotely, via long-range fiber connections. Aikeke information criterion (AIC) is reported for each model in brackets. There is no difference between models in amyloid fits. For tau and atrophy, the lowest overall AIC (**boldface**), and highest R (**boldface**), are achieved by the 1-way interaction from amyloid to tau aggregation; consequently it is highlighted by green box around it. The remote-interaction and 2-way interaction models are insufficiently different from this 1-way model. Comparisons between these models via Fisher’s t-test are contained in Table S3.

**Table 3:** Pearson’s R of correlation between alternative spread models and empirical regional statistics.

Empirical data	*Aβ* → *tau* interaction, Connectivity	Fiber Distance	Fisher R-to-z, Fiber vs C	Euclidean Distance	Fisher R-to-z, Euclidean vs C
EMCI Aβ	0.71	0.56	**p < 0.01**	0.57	**p < 0.01**
LMCI Aβ	0.49	0.43	p = 0.10	0.42	p = 0.09
AD Aβ	0.70	0.56	**p < 0.01**	0.57	**p < 0.01**

EMCI tau	0.48	0.25	**p < 0.01**	0.24	**p < 0.01**
LMCI tau	0.75	0.31	**p < 0.01**	0.35	**p < 0.01**
AD tau	0.73	0.34	**p < 0.01**	0.36	**p < 0.01**
EMCI atrophy	0.35	0.00	**p < 0.01**	0.18	**p = 0.03**
LMCI atrophy	0.62	0.29	**p < 0.01**	0.41	**p = 0.025**
AD atrophy	0.66	0.17	**p < 0.01**	0.22	**p < 0.01**

Fisher’s R-to-z transform was computed between the connectome and the alternative modes of spread, and its p-value is shown alongside. The connectome-mediated model significantly outperforms the two distance-based models (p values denoted in red font).
